# Supplementary material for: Efficacy of oxfendazole treatment against infective larvae of Litomosoides sigmodontis
Source: PLoS Negl Trop Dis. 2026 Apr 21;20(4):e0014268. doi: 10.1371/journal.pntd.0014268 (PMC13128108; doi:10.1371/journal.pntd.0014268)
Supplement: Attachment — Submitted filename: Response to Reviewers final.docx [file pntd.0014268.s002.docx]

Institute of Medical Microbiology, Immunology and Parasitology (IMMIP)

University Hospital Bonn, Venusberg-Campus 1, 53127 Bonn

9. April 2026

**Response to reviewers for the manuscript “Efficacy of oxfendazole treatment against infective larvae of *Litomosoides sigmodontis*”**

We thank the reviewers for their work and suggestions and the editorial team for their consideration. We have made the suggested changes wherever possible and hope that this revised manuscript meets the requirements for publication.

Best wishes,

Marc Hübner

**Journal Requirements:**

1) We ask that a manuscript source file is provided at Revision. Please upload your manuscript file as a .doc, .docx, .rtf or .tex. If you are providing a .tex file, please upload it under the item type u2018LaTeX Source Fileu2019 and leave your .pdf version as the item type Manuscript.

**Answer**: A revised version of the manuscript has been submitted as a .docx file.

2) Please upload all main figures as separate Figure files in .tif or .eps format. For more information about how to convert and format your figure files please see our guidelines: 
<https://journals.plos.org/plosntds/s/figures>

**Answer**: The figures are uploaded as .tif files (“Figure 1” and “Figure 2”).

3) We note that your Data Availability Statement is currently as follows: "All original data will be provided as excel sheet". Please confirm at this time whether or not your submission contains all raw data required to replicate the results of your study. Authors must share the “minimal data set” for their submission. PLOS defines the minimal data set to consist of the data required to replicate all study findings reported in the article, as well as related metadata and methods (https://journals.plos.org/plosone/s/data-availability#loc-minimal-data-set-definition).
For example, authors should submit the following data: 
- The values behind the means, standard deviations and other measures reported;
- The values used to build graphs;
- The points extracted from images for analysis..
Authors do not need to submit their entire data set if only a portion of the data was used in the reported study.
If your submission does not contain these data, please either upload them as Supporting Information files or deposit them to a stable, public repository and provide us with the relevant URLs, DOIs, or accession numbers. For a list of recommended repositories, please see https://journals.plos.org/plosone/s/recommended-repositories. 
If there are ethical or legal restrictions on sharing a de-identified data set, please explain them in detail (e.g., data contain potentially sensitive information, data are owned by a third-party organization, etc.) and who has imposed them (e.g., an ethics committee). Please also provide contact information for a data access committee, ethics committee, or other institutional body to which data requests may be sent. If data are owned by a third party, please indicate how others may request data access.

**Answer**: All data is included in the figures in the submitted and revised version of the manuscript. In addition, the raw data that was used to generate the figures is submitted in an excel sheet named “Raw data Oxf Lito L3 short report” for better reproducibility.

4) Please amend your detailed Financial Disclosure statement. This is published with the article. It must therefore be completed in full sentences and contain the exact wording you wish to be published.
- State the initials, alongside each funding source, of each author to receive each grant. For example: "This work was supported by the National Institutes of Health (####### to AM; ###### to CJ) and the National Science Foundation (###### to AM)."
- State what role the funders took in the study. If the funders had no role in your study, please state: "The funders had no role in study design, data collection and analysis, decision to publish, or preparation of the manuscript.".
If you did not receive any funding for this study, please simply state: The authors received no specific funding for this work.

**Answer:** The financial disclosure statement has been amended.

**Reviewers' comments:**

Reviewer #1: The Methods are well-described and appropriate for the aims of the study.

Reviewer #2: The paper describes that Oxfendazole exhibits a robust efficacy against the L3 larval stage of Litomosoides sigmodontis in mice. This work supports the ongoing clinical development of Oxfendazole as a broad stage filaricide/nematicide. The benzimidazole originates from veterinary medicine and is now repurposed for human use. While the team showed in previous work, that Oxfendazole showed macrofilaricidal efficacy, but no direct effect on microfilariae, only this manuscripts demonstrates that Oxfendazole is active against L3 larvae.
The experimental design is sufficiently described and scientifically sound:
• BALB/cJ mice were naturally infected with L. sigmodontis via exposure to infected mites.
• Drug was administered orally twice daily, starting 1 day post infection, for 3 or 5 days.
• Doses tested: 5 mg/kg and 12.5 mg/kg.
• Worms were recovered and measured 35 days post infection.

**Results**


-Does the analysis presented match the analysis plan?

-Are the results clearly and completely presented?

-Are the figures (Tables, Images) of sufficient quality for clarity?

Reviewer #1: The Results are clear and the analyses justified and appropriate.

Reviewer #2: Key Findings
1. Strong reduction in worm burden
• 5 day treatment at 12.5 mg/kg: → 97.6% median reduction in total worms → Female worms reduced 100%, males 95.7%
• 3 day treatment showed dose dependent efficacy: → 12.5 mg/kg for 3 days reduced total worms by 82.6% → 5 mg/kg for 3 days had only modest effect (~30% reduction)
2. Worm development was impaired
• Surviving worms were significantly shorter, especially after 5 day treatment.
• Oxfendazole is highly effective against the infective L3 stage of L. sigmodontis.

**Conclusions**


-Are the conclusions supported by the data presented?

-Are the limitations of analysis clearly described?

-Do the authors discuss how these data can be helpful to advance our understanding of the topic under study?

-Is public health relevance addressed?

Reviewer #1: The regimen investigated here was clearly highly effective against L3 stage parasites. My concern is the seemingly minor relevance. To be useful, one would either have to maintain constant plasma levels in people living in endemic regions or treat individuals who were briefly exposed to transmission. Activity against L4 and L5 stages might be more clinically relevant (not considering macrofilaricidal activity, which has been reported).

**Answer**: We thank the reviewer for their insightful comment. We fully agree, that demonstrating efficacy against the further developing stages would elevate the impact of the study. However, due to the constraints of the project (both time and budget), we are unable to perform the additional required studies in the foreseeable future.

Reviewer #2: I would encourage the authors to extend the background and discussion about animal health as well. To prevent canine heartworm disease, the migrating larvae need to be treated. This could also lead to an outlook on what the treatment against L4 larvae would look like. Furthermore, the authors may want to discuss the dose-response comparing the larvicidal versus the adulticidal effects to a certain degree (particularly as parasite isolate, mouse strain and drug are uniform).
Minor remark: The graphs and abstract do not clearly state that the treatment is 2 times per day.

**Answer**: Additional information about the use of oxfendazole in the veterinary field and the comparison of the dose-response versus the different stages has been added. In addition, the graphs have been adjusted to clarify the treatment regimen.

**Editorial and Data Presentation Modifications?**

Use this section for editorial suggestions as well as relatively minor modifications of existing data that would enhance clarity. If the only modifications needed are minor and/or editorial, you may wish to recommend “Minor Revision” or “Accept”.

Reviewer #1: A minor concern: the authors should stress that the PK properties of the drug are atypical for benzimidazole anthelmintics and enable much higher systemic exposure than attained with albendazole, for example, which is included in combination therapy for LF and has been investigated for other systemic helminth infections, including as a way to reduce microfilaremia in Loa infections. Thus, one would anticipate higher efficacy with oxfendazole, assuming intrinsic potency is similar to other BZs.

**Answer**: The reviewer’s expertise with benzimidazoles is greatly appreciated and additional discussion about the PK properties including references to the recent publication by Assmus et al. concerning the PK profile of oxfendazole in healthy African volunteers has been added to the discussion in line 202ff.

Reviewer #2: I would encourage the authors to extend the background and discussion about animal health as well. To prevent canine heartworm disease, the migrating larvae need to be treated. This could also lead to an outlook on what the treatment against L4 larvae would look like. Furthermore, the authors may want to discuss the dose-response comparing the larvicidal versus the adulticidal effects to a certain degree (particularly as parasite isolate, mouse strain and drug are uniform).
Minor remark: The graphs and abstract do not clearly state that the treatment is 2 times per day.

**Answer**: Additional information about the use of oxfendazole in the veterinary field and the comparison of the dose-response versus the different stages has been added. In addition, the graphs have been adjusted to better clarify the treatment regimen.

**Summary and General Comments**


Use this section to provide overall comments, discuss strengths/weaknesses of the study, novelty, significance, general execution and scholarship. You may also include additional comments for the author, including concerns about dual publication, research ethics, or publication ethics. If requesting major revision, please articulate the new experiments that are needed.

Reviewer #1: The study is properly and carefully done. Really, my only concern is its relevance for therapy.

Reviewer #2: This is a well‑executed and valuable study that provides the first in vivo evidence of Oxfendazole’s efficacy against infective larvae of Litomosoides sigmodontis. The results are clear, the methodology is appropriate, and the findings significantly advance the understanding of Oxfendazoles therapeutic spectrum potential.

With minor revisions to strengthen context and clarity, the manuscript is well suited for publication.

**Answer**: We thank the reviewer’s for their positive feedback.

PLOS authors have the option to publish the peer review history of their article ([what does this mean?](https://track.editorialmanager.com/CL0/https:%2F%2Fjournals.plos.org%2Fplosntds%2Fs%2Feditorial-and-peer-review-process%23loc-peer-review-history/1/010f019c65d84429-8447c9fb-a444-4da5-bd17-ee286057db32-000000/zwamaK3zFW2oqww0x5Rhjdu2NhAuJZx9f32Lp7TDYSs=250)). If published, this will include your full peer review and any attached files.


If you choose “no”, your identity will remain anonymous but your review may still be made public.


**Do you want your identity to be public for this peer review?** For information about this choice, including consent withdrawal, please see our [Privacy Policy](https://track.editorialmanager.com/CL0/https:%2F%2Fwww.plos.org%2Fprivacy-policy/1/010f019c65d84429-8447c9fb-a444-4da5-bd17-ee286057db32-000000/8vN4JT0GvBCGyC1ZwJlroEqIM3UadfysIG4UqpAZgjI=250).

Reviewer #1: No

Reviewer #2: **Yes:** Daniel Kulke
